# Statistical emulation of landslide-induced tsunamis at the Rockall Bank, NE Atlantic

**DOI:** 10.1098/rspa.2017.0026

**Published:** 2017-04-12

**Authors:** D. M. Salmanidou, S. Guillas, A. Georgiopoulou, F. Dias

**Affiliations:** 1 School of Mathematics and Statistics, University College Dublin, Dublin, Ireland; 2 Earth Institute, University College Dublin, Dublin, Ireland; 3 School of Earth Sciences, University College Dublin, Dublin, Ireland; 4 Department of Statistical Science, University College London, London, UK

**Keywords:** landslide tsunamis, Bayesian calibration, statistical emulators

## Abstract

Statistical methods constitute a useful approach to understand and quantify the uncertainty that governs complex tsunami mechanisms. Numerical experiments may often have a high computational cost. This forms a limiting factor for performing uncertainty and sensitivity analyses, where numerous simulations are required. Statistical emulators, as surrogates of these simulators, can provide predictions of the physical process in a much faster and computationally inexpensive way. They can form a prominent solution to explore thousands of scenarios that would be otherwise numerically expensive and difficult to achieve. In this work, we build a statistical emulator of the deterministic codes used to simulate submarine sliding and tsunami generation at the Rockall Bank, NE Atlantic Ocean, in two stages. First we calibrate, against observations of the landslide deposits, the parameters used in the landslide simulations. This calibration is performed under a Bayesian framework using Gaussian Process (GP) emulators to approximate the landslide model, and the discrepancy function between model and observations. Distributions of the calibrated input parameters are obtained as a result of the calibration. In a second step, a GP emulator is built to mimic the coupled landslide-tsunami numerical process. The emulator propagates the uncertainties in the distributions of the calibrated input parameters inferred from the first step to the outputs. As a result, a quantification of the uncertainty of the maximum free surface elevation at specified locations is obtained.

## 1. Introduction

Nowadays, submarine landslides are recognized as one of the principal tsunami-triggering mechanisms [1–3]. Marine surveys conducted during the last century have shed light on the extensive submarine landslide deposits lying on the seabed of continental slopes and the slopes of volcanic islands [4,5]. Known examples of tsunamigenic landslides in the North Atlantic Ocean have been extensively studied in the past decades under the framework of numerical models [6–13]. The characteristics of landslide tsunamis may vary depending on the type of the slide mechanism and the slide parameters [14]. Some critical parameters affecting tsunami generation are the volume of the sliding material, the nature of the failure mechanism, the initial acceleration and maximum velocity of the landslide [1,3,14]. The complexity of submarine landslides and the lack of direct observations add to the uncertainty of the related tsunami hazard and render the modelling of the phenomenon a complicated task.

The numerical codes used for landslide and tsunami modelling may vary depending on several features like the physics, the numerical scheme and the dimensions [15]. The shallow water theory constitutes the most common theory that has been applied so far in the research of landslide tsunamis [15]. Despite the simplifications in the physics, the applicability of the shallow water equation (SWE) solvers renders them a popular choice [15]. Known SWE solvers have been used for simulations of landslide tsunamis [16–18] and there are many other events where the shallow water theory was employed [7–9,16,19–21]. Nonetheless, in cases where the frequency dispersion of the landslide tsunami becomes important, the SWE solvers do not capture some of the changes in the wave characteristics [22].

To account for dispersive effects during landslide tsunami propagation, the Boussinesq approximation can be employed and state-of-art Boussinesq solvers have been used for operational tsunami research [11,17,23,24]. To capture adequately the interaction between the landslide and the water, numerical codes that solve the three-dimensional Navier–Stokes (NS) equations or the three-dimensional potential flow equations can also be used [25–27]. NS codes are especially useful for the modelling of sub-aerial landslide tsunamis where the physical processes can become very complex. Albeit, the high computational costs of the NS solvers may render their applicability challenging [15]. To counterbalance for that, NS solvers can be used in conjunction with Boussinesq solvers to study the far-field potential of landslide tsunamis [11,12,28,29].

In cases where large numbers of numerical simulations become cumbersome, statistical techniques can be employed to shed light on the way that the mechanical processes can influence the results. Up to present, various statistical methods have been implemented to quantify and minimize the degree of uncertainty in tsunami science [30]. The use of statistical emulators, also referred to as statistical surrogates, in place of the deterministic codes, constitutes a prominent solution [30]. Statistical emulators actually form stochastic representations of the deterministic computer models used to simulate a physical process. The objective of the emulators is not to entirely replace the deterministic codes but to act in a complementary way, by assessing the results of numerous scenarios in only a few moments of time. As tsunami models can be computationally expensive to run, building statistical surrogates can be used instead, to assess uncertainty and conduct sensitivity analyses in shorter computational times.

Emulators lead to inexpensive probabilistic predictions of the examined system and contribute towards a better understanding of the system's behaviour. Some recent examples of building statistical surrogates of tsunami numerical codes exist in the literature [31–33]. Sarri *et al.* [31] have built a statistical surrogate of the analytical model for landslide tsunamis developed by Sammarco & Renzi [34]. The emulator was constructed using a Gaussian Process (GP) and was validated with the Leave-One-Out diagnostics (LOO). Sraj *et al.* [32] used Polynomial Chaos (PC) methods to build a statistical surrogate based on measurements of the free surface elevation for the 2011 Japanese tsunami and quantify the uncertainty in bottom friction parametrization. Beck & Guillas [33] developed an algorithm for sequential experimental design to efficiently build the statistical surrogate of a tsunami code.

**Figure 1.**
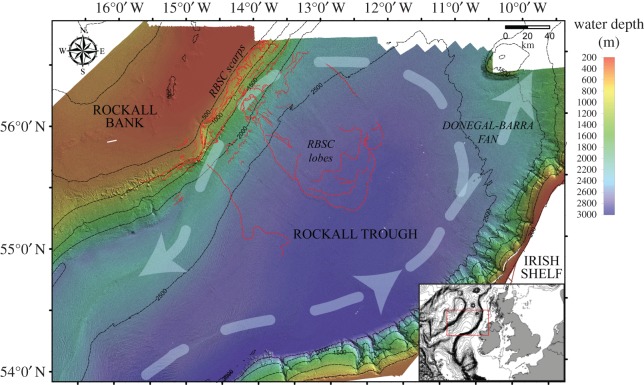
Multibeam bathymetry map of the Rockall Trough based on the Irish National Seabed Survey (INSS) dataset. The RBSC scarps and lobe limits are indicated with red lines. Arrows show the general oceanographic circulation. Bathymetric contours are shown with thin black lines. The map on the right-bottom corner shows the position of the RBSC with respect to Ireland and the UK. Adapted from Georgiopoulou *et al.* [35]. (Online version in colour.)

The tsunami mechanism under study in this work is the slope failure at the Rockall Bank Slide Complex (RBSC), a region of extensive submarine landslide deposits, in the Northeast Atlantic Ocean (figure 1). To simulate submarine sliding and tsunami generation in the region, the landslide code VolcFlow [36] and the tsunami code VOLNA [37] are used. The statistical emulation of the one-way coupled numerical process is ultimately performed to quantify uncertainties. The numerical codes imply simplifications that should be further explored for a comprehensive geophysical analysis of the event. The main objective of this paper is to demonstrate the applicability of statistical emulation to assess thousands of otherwise computationally intensive simulations in a realistic setting, rather than claiming generality of the hazard assessment.

We concentrate our attention on statistical methods as a means to enhance the numerical simulations of a real event, with Rockall Bank as an illustration. In particular, we incorporate data of ongoing research on the landslide deposits in order to assess the uncertainties of the landslide characteristics, this is done with the Bayesian calibration. To our knowledge, this is the first use of Bayesian calibration in deposit modelling. With the aid of the built-in emulator, we can finally make predictions of the maximum tsunami amplitudes that result from varying landslide scenarios and quantify the uncertainty of the tsunami amplification.

Prior to the propagation of uncertainties through statistical emulation, the uncertainties in the landslide characteristics need to be estimated by comparing modelled deposits against observations. The objective is to infer the characteristics of the landslide that yield the observed run-out distance, the inference of other tsunamigenic characteristics unrelated to the parameters present in this study is not possible here. The Bayesian calibration used here is based on the framework of Kennedy & O’Hagan [38]. We follow a similar approach to the one described in Guillas *et al.* [39], where the statistical surrogate of a computational fluid dynamics (CFD) model was calibrated against observations. In this Bayesian framework, Markov Chain Monte Carlo (MCMC) methods and the Metropolis–Hastings algorithm [40] are utilized to find the optimal ranges, and distributions over these ranges, of the parameters.

To propagate the uncertainty from the landslide characteristics identified in the calibration to the resulting tsunamis, we build a statistical emulator of the combined landslide–tsunami numerical process, based on a GP. The prediction as well as the associated uncertainties are performed using the kriging equations. The emulator enables us to make predictions of the maximum tsunami wave amplitudes that would result from different sets of input landslide parameters, in a computationally fast, efficient and inexpensive way. Indeed, the possible values of the calibration parameters, i.e. their distributions are fed into the emulator to produce the probabilistic distributions of the maximum tsunami wave amplitudes at specified locations.

## 2. Submarine sliding in the Rockall Bank Slide Complex

The RBSC is located 350–400 km offshore the northwest Irish shoreline, in the NE Atlantic Ocean (figure 1). The failure escarpments of the complex are identified on the eastern margin of the Rockall Bank, an almost flat-topped, underwater plateau (figure 1). The depositional area of the RBSC spreads on the seabed of the Rockall Trough, a steep-sided elongate depression 300–3000 m deep in the study area. The complex forms the largest region of submarine slope failure scarps in the Irish Atlantic margin, exhibiting an irregular width-to-length aspect ratio of 120×150 km [41].

Until recently, the RBSC had been considered as one single event mass flow [41]. New sedimentary and seismic data taken from the depositional lobes of the RBSC demonstrate that the RBSC morphology is the result of multiple phases of slope failure, at least three, that were separated by long periods of slope stability [42]. Radiocarbon dating showed that the most recent event took place 21 ka, which is 3000 years post Last Glacial Maximum (LGM), when the British Irish Ice Sheet (BIIS) was still at maximum extent and was only just starting to destabilize [42]. One hypothesis for the triggering mechanism suggests that sedimentation on the upper part of the slope enhanced by strong bottom current activity removed support from the base-of-slope, leading to slope instability and subsequently failure [35,41]. Updated research supports that bottom currents would have still been weak at the time of the slide and therefore an earthquake is considered as the most likely triggering mechanism [42].

Reconstruction of the preslide slope morphology yielded a sediment volume of 265–765 km^3^ [35]. The slope was divided into two main areas of failure scarps: the upper and the lower slope regions [35]. Based on an early interpretation of seismic data collected in the area in 2011 [43] and the INSS bathymetry at least three phases of collapse were distinguished, with the most recent episode initiating from the lower slope region. The volume of the event was estimated to be approximately 400 km^3^. The continuous activity of contour currents in the area implies that the slope scars may have formed rapidly, due to a large part of the slope collapsing simultaneously [41].

The failure mechanism and the large volume of the sediments raise questions for the tsunamigenic potential of the failure. Any generated tsunamis, chronologically near the LGM, would probably not have affected Ireland, because the western shelf and coast were still covered by ice [44]. Nevertheless, the tsunamigenic potential of similar volume sliding episodes nowadays could pose a significant risk to the Irish shoreline. To address this possibility and the magnitude of the generated tsunamis, some preliminary numerical simulations were conducted, focusing on the modelling of the event [45]. The results show that sliding in the RBSC can lead to tsunami generation of an uncertain size as parametric variability is large and thus requires tightening via calibration against observations as we do here. For the parameter calibration and for the set-up of the statistical emulator, we turn our attention to numerical simulations of landslide initiation and tsunami generation in the lower slope region. We use as reference the depositional area of the latest episode as it forms the most voluminous and chronologically most recent failure episode in the RBSC.

## 3. Numerical algorithms

To study submarine sliding and tsunami generation in the RBSC, the one-way coupling of two numerical models is performed. We make use of the VolcFlow code [36] for the landslide modelling. The code is not too computationally demanding and has been efficiently used to study several cases. The VOLNA code [37] is then used for the simulations of the landslide tsunamis. Both codes solve the depth-averaged shallow water equations and can thus be considered as simplified representations of reality when compared with more sophisticated codes. The rationale behind the choice of the two codes is their feasibility in the two-dimensional domain.

The two-dimensional code VolcFlow was initially developed for the simulation of dense-isothermal volcanic-geophysical flows [36]. The code has been used to model various situations ranging from rock avalanches [36] and pyroclastic surges [46] to submarine landslides and tsunami generation [20]. VolcFlow is a finite-difference code that solves the depth-averaged equations of mass and momentum using a shock-capturing numerical method based on a double upwind Eulerian scheme [36]. For the full derivation of the equations that govern landslide motion and deposition in VolcFlow we refer the reader to [36].

In VolcFlow, the sliding mass spreads during motion and the landslide deposition follows the topography of the grid [36].The code also accounts for the simulation of various rheological flow regimes [47]. Common regimes to simulate sliding in the submarine domain are the Herschel–Bulkley and the Bingham rheology or an extension of it [48–50]. A simplified Bingham rheology can be modelled with the code where neither layering nor additional mechanisms such as hydroplanning and remoulding are incorporated. Based on the selection of the input parameters for the calculations of the shear retarding stress, the flow properties can vary representing different flow types. For our cases, we make use of a rheological model that incorporates the viscoplastic and frictional properties of the sliding material plus a velocity-dependent term. We use the Cartesian coordinate system, where the *x*- and *y*-axes represent the EW and NS horizontal directions, respectively, and the *z*-axis the vertical direction. The retarding stress **T**=(*T*_*x*_,*T*_*y*_) is then given by

3.1 T=T0u∥u∥+μdudh+ρh(g cos ⁡φ+∥u∥2r)tan ⁡ϕbedu∥u∥+ξρ∥u∥u,

where *T*_0_ is the yield strength, **u**(*u*_*x*_,*u*_*y*_) the depth-averaged velocity, *μ* the dynamic viscosity, d**u**/d*h* the shear rate, *ρ* the apparent density of the mixture, *h*(*x*,*y*) the slide thickness, *g* the acceleration due to gravity, *φ* the slope angle, *r* the slope curvature, *ϕ*_bed_ the basal friction angle and ξ a non-dimensional coefficient used to represent the effect of turbulence and/or collisions.

The first three terms of the equation resemble the model introduced by Norem *et al.* [51] to describe the mobility of subaqueous flows. The last term of the equation is making use of the coefficient of turbulence introduced by Voellmy [52]. This term can be useful in the modelling of submarine landslides as ξ can account for the hydrodynamic drag, one of the most critical forces resisting motion in the submarine domain [14]. The added mass coefficient, which also forms an important parameter affecting landslide acceleration and tsunamigenesis [9,14,53], was not incorporated in this study. As we perform one-way coupling with the tsunami model, to partially account for motion in the submarine domain we incorporate the drag term in addition to a reduced landslide density (§5). Preliminary simulations show that neglecting or choosing very small values for ξ results in large peak velocities of the landslide and consequently high free surface elevations [45].

We model tsunami generation, propagation and run-up with VOLNA. The current version of the code can be run on both CPU and GPU processors. The Nonlinear Shallow Water Equations (NSWE) are solved with a finite volume method. To capture the complexity of the bathymetry and the surrounding coastlines in a specified location, the code employs unstructured triangular meshes. VOLNA has been efficiently used to model tsunami generation in different cases and in various settings [45,54,55]. For landslide tsunami propagation, dispersive effects are not accounted for by the code, a brief discussion is given in §6c. The exposition of the equations and in depth details about the numerical methodology in VOLNA can be found in [37].

## 4. Statistical calibration

In numerical modelling, the issue of assigning the appropriate parameter values to model a physical process forms a common challenge. Statistical calibration can reduce the uncertainty in the input parameters by confronting model outputs to observations [38]. In particular, Bayesian calibration allows the acknowledgement of all sources of uncertainty (typically model inadequacies, observation errors) and enables experts to encapsulate current knowledge in prior distributions of the parameters. Posterior distributions of the input parameters are then obtained in the Bayesian paradigm. The inference of the parameters’ posterior distributions is carried out by balancing prior information, reflecting the current knowledge, against the information from the observations. The statistical approach introduced by Kennedy & O’Hagan [38] is the mainstream method used to perform a Bayesian calibration of a deterministic code, which we follow here.

Let us denote the true physical system, *ζ*(*x*), with *x* the observable inputs. The input parameters can be subdivided into two groups: the inputs *x* whose values are observed (e.g. location, conditions) and thus known, and the calibration parameters *θ* whose values are unobserved, and thus unknown (typically numerical constants required for the simulations, such as initial conditions and parameters of physical processes). To accurately simulate the physical system in the future, we must first estimate the optimal values of the unknown calibration parameters. As for each numerical experiment both parameter groups have to be specified, the observable inputs are assigned values of *x*=(*x*_1_,…,*x*_*m*_) and the calibration parameters are assigned values of *I*=(*I*_1_,…,*I*_*m*_), where *x* and *I* are known, and m stands for the total number of the numerical computations. The computed output from the simulations can then be written as a function of the input variables and the calibration parameters

4.1 $$Y_{i}^{C}=\eta (x_{i},I_{i})+e_{\eta },$$

where $Y_{i}^{C}$ is the output of the code and *η*(*x*_*i*_,*I*_*i*_) the expected output (these would be equal if the results were not subject to intrinsic numerical error, *e*_*η*_, typically small). For an appropriate choice of *I*=*θ* the numerical output *η*(*x*_*i*_,*θ*) would be the best numerical representation of the physical process, but can still be different from it due to a discrepancy caused by the lack of physical representation in the model or a low resolution for instance. The relationship between the *n* observations of the physical system in the field $Y_{i}^{F}=(Y_{1}^{F},\ldots ,Y_{n}^{F})$ and the model outputs *η*(*x*_*i*_,*I*_*i*_) can be expressed as

4.2 $$Y_{i}^{F}=\zeta (x_{i})+e_{i}=\eta (x_{i},\theta )+\delta (x_{i})+e_{i},$$

where *e*_*i*_ is the observation error at the *i*th observation and *δ*(*x*_*i*_) denotes the bias, or model inadequacy function, meaning the discrepancy between the optimal value of the physical process and the computer output when the inputs are assigned optimal values. Note that, due to the bias, the deterministic code can never truly reproduce the physical system, even if the best values of the parameters were used for the simulations. Accounting for model inadequacy is, thus, of critical importance as it helps avoiding a possible overestimation or underestimation of the calibration parameters when the statistical model tries to unreasonably fit the results of the computations with the observations. To perform the calibration, prior information for the calibration parameters is required. It is derived from previous field experiments, general scientific knowledge or numerical simulations, and represented in the form of prior distributions on these parameters.

For two vectors, *x* and *I*, which belong to a computational design *D^M^*, the simulator output at the design points is defined as *η*(*x*,*I*). If *x*∈*R*^*p*^ and *I*∈*R*^*l*^, then the function *η*(.,.) maps *R*^(*p*+*l*)^ to *R*. The output of the code is unknown for inputs that differ from the specified design points and therefore, has to be approximated. To do so, we assume that the unknown function is a GP, and its outputs are realized as such. The random function is certain at the design points and uncertain at other points.

To specify a GP prior model for *η*(*x*,*I*), a mean function *μ*(*x*,*I*) and a covariance function *Cov*((*x*,*I*),(*x*′,*I*′)) are required. We choose the commonly used product exponential covariance function. It is given by a product of individual terms, per parameter, of the form $\exp(-\gamma_{k}{|x_{k}-x_{k}^{\prime }|}^{\alpha })$ for *k*=1,…,*p*, and $\exp(-\gamma_{k}{|I_{k}-I_{k}^{\prime }|}^{\alpha })$ for *k*=*p*+1,…,*p*+*l*. The *γ*'s act as correlation lengths parameters because they characterize the strength of the relationship between outputs with respect to the proximity between inputs. The covariance function is reparametrized for computational convenience in the following form:

4.3 $$\mathrm{Cov}((x,I),(x^{\prime },I^{\prime }))=\frac{1}{\lambda_{\eta }}\prod_{k=1}^{p}{\left(\rho_{k}^{\eta }\right)}^{2{|x_{k}-x_{k}^{\prime }|}^{\alpha }}\times \prod_{k=1}^{l}{\left(\rho_{p+k}^{\eta }\right)}^{2{|I_{k}-I_{k}^{\prime }|}^{\alpha }},$$

with $\rho_{k}^{\eta }=\exp\left(-\beta_{k}^{\eta }/4\right)$, where the (*p*+*l*)-vector *ρ*^*η*^ controls the strength of the dependence in each of the component directions of *x* and *I*; λ_*η*_ and *β*^*η*^ are the precision and correlation hyperparameters of *η*(.,.), and *α* controls the smoothness of *η*(.,.). For a smooth representation of the results, we use *α*=2; smaller values of *α* result in rougher representations, and might be beneficial in some cases (combination of analysis and experience in other settings [39] did not show any benefit here).

A GP model is also specified for the discrepancy term *δ*(*x*_*i*_). The observation error *e*_*i*_ and the numerical error *e*_*η*_ are modelled as independent normal distributions: *e*_*i*_∼*N*(0,1/λ_*e*_) and *e*_*η*_∼*N*(0,1/λ_*e*_*η*__), where λ_*e*_ and λ_*e*_*η*__ are the respective precision hyperparameters for *e*_*i*_ and *e*_*η*_. Finally, the likelihood for a *n*+m-joint vector with values *Y* ^F^=(*x*_1_,*θ*),…,(*x*_*n*_,*θ*) for its first *n* components and *Y* ^*C*^=(*x*_1_,*I*_1_),…,(*x*_*m*_,*I*_*m*_) for its final *m* components is given by a multivariate normal distribution

4.4 $$\left(\begin{array}{c} Y^{F}\\ Y^{C} \end{array}\right)=MVN(0,\Sigma_{y}),\;$$

where

4.5 $$\Sigma_{y}=\Sigma_{\eta }+\left(\begin{array}{cc} \Sigma_{\delta } & 0\\ 0 & 0 \end{array}\right)+\left(\begin{array}{cc} \Sigma_{e} & 0\\ 0 & \Sigma_{e_{\eta }} \end{array}\right),$$

where *Σ*_*η*_ is the product exponential correlation matrix for *η*(*x*,*I*) from (4.3), *Σ*_*δ*_ is the product exponential correlation matrix for *δ*(*x*_*i*_), similarly derived, and *Σ*_*e*_, *Σ*_*e*_*η*__ are the independent normal noises for the observation and the numerical errors. In this study, we have *n*=9 field observations of the landslide extent and m=100 numerical experiments.

A mean function of *μ*=0 is employed as preliminary analyses showed no benefits in using more advanced functions here. To standardize the entire set of the responses, the variability in the simulator (1/λ_*η*_) is set to 1. The design space for the observable inputs is scaled to [0,1]^*p*^, and the design space for the calibration parameters is scaled to [0,1]^*l*^. To complete the Bayesian formulation, independent prior distributions *π*(.) are specified for each of the parameters of (4.3), for *η*(.,.) and similarly for *δ*(.). A MCMC method can then be employed to explore the posterior distributions and estimate the calibration parameters. The model makes use of the Metropolis–Hastings algorithm which generates a sequence of samples [40].

In order to obtain the posterior distributions, prior assumptions for the distributions of the parameters are required. For the calibration parameters *θ*, we define a prior parameter range, based on the literature and past numerical simulations, and perform Latin Hypercube Sampling (LHS) to select the values for the numerical experiments. We specify the prior distributions of the correlation and precision hyperparameters following the approach of Guillas *et al.* [39]. The prior distributions of the correlation hyperparameters $\beta_{k}^{\eta }$ and $\beta_{k}^{\delta }$ are selected so that the expected values of the corresponding correlations (*ρ*_*η*_ and *ρ*_*δ*_) are lower than 1 (resulting in an insignificant effect). The precision hyperparameters λ_*η*_, λ_*δ*_, λ_*e*_ and λ_*e*_*η*__ are assigned *Γ* prior distributions of λ_*η*_∼*Γ*(10,10) (as the set of the responses is standardized λ_*η*_ approximates 1), λ_*δ*_∼*Γ*(10,0.3), λ_*e*_∼*Γ*(10,0.03) and λ_*e*_*η*__∼*Γ*(10,0.001) meaning that the standard deviation is expected to be 3%, 0.3% and 0.01% of the standardized responses. Note that very recent papers have mentioned the challenges and issues in this Bayesian calibration set-up, with possible generic solutions [56,57].

Based on the prior information and the MCMC implementation, posterior distributions are sampled numerically for all the calibration parameters. The posterior distributions will be used as a tool to reach conclusions for the optimal values of the parameters and quantify their uncertainties. The posterior realizations of the parameters, characterizing empirically the distributions, will be utilized in the next step, which is to propagate these uncertainties in the resulting tsunami wave amplitudes through the use of a statistical surrogate of the tsunami model.

## 5. Ranges of the calibration parameters

First, we allocate prior distributions regarding the unknown input parameters *θ*, as a necessary step for the calibration. In this study, we select six calibration parameters to vary, one is geomorphological and the other five rheological. From a geomorphological perspective, the maximum thickness of the landslide, $h_{\max}$, at *t*=0 s considers the headwall scarp height and the thickness of the slide to be the same. Consequently, this has an impact on the volume, *V* , of the landslide. The rheological parameters are: the yield strength of the landslide, *T*_0_, the basal friction angle, *ϕ*_bed_, the dynamic viscosity, *μ*, and the apparent density, *ρ*. Finally, we introduce and vary the coefficient of turbulence, ξ, which is used to represent the hydrodynamic drag.

The slide thickness range derives from measurements of the scarp heights on the slope region. The lower slope region is dominated by smaller scarp heights ranging between 60 and 120 m [35]. The scarp height distribution of each event comes in contrast with the attributed volume distribution, which possibly indicates that the landslide was of an erosive nature and had embodied a large amount of the basal sediments during motion. As such complex flow behaviour cannot be easily captured numerically, in our simulations we model the initial volume of the landslide to represent the estimated volume of the event. We simulate a landslide of Gaussian shape, keeping the spatial extent fixed to match closely the extent of the scarps on the slope. The width-to-length aspect ratio of the landslide is 0.84, a range of $h_{\max}=60$–120 m is selected for the maximum thickness of the landslide corresponding, respectively to a volume range of *V* =198–396 km^3^.

As measurements for the rheological parameters are not available, the selection of the input ranges was mostly based on the literature and the results of numerical experiments [45]. The yield strength of large-scale muddy debris flows in the submarine domain may vary between 4000 and 15 000 Pa [50]. Numerical simulations of clay and silt-rich landslides have used a yield strength varying between 10 000 and 25 000 Pa [58]. Elverhoi *et al.* [59] have provided numerical simulations of the less cohesive (low clay sediment content) Grand Banks landslide with yield strengths of 3000 Pa. The best-fit solution in the simulations of the BIG’95 debris flow results from a flow with yield strength of 800 Pa, possibly due to the effect of hydroplanning or entrapment of mobile materials under the flow [60]. The current numerical simulations show that small yield strengths are required for the simulated flow to reach the observed run-out distance. A range of 100–10 000 Pa was initially selected for the yield strength of the landslide. After the first batch of numerical experiments, the range of the yield strength was further reduced to 100–5000 Pa in an attempt to optimize the results of the simulations by generating deposits that were in agreement with the observed deposits and decrease the computational time.

Common values of basal friction angles of underwater landslides may range from 0^°^ to 5–7^°^ (for purely frictional models) [20]. Preliminary results of the numerical simulations in the RBSC show that when the coefficient of turbulence is negligible, the best fit values for the approximation of the run-out distance vary from 0^°^ (for a Bingham fluid behaviour) to approximately 1.3^°^ (for less cohesive landslides); apart from the yield strength the values of the other parameters do not affect as significantly the flow run-out. A range of 0–1^°^ was initially selected for the basal friction. The range was further reduced to *ϕ*_bed_=0.0–0.5^°^ for similar reasons as in the case of the yield strength.

Numerical simulations of submarine flows indicate that when the landslide material is characterized by high yield strength, the effect of the dynamic viscosity on the velocity and run-out distance of the landslide during the post-failure stage is negligible [61,62]. Dynamic viscosity may have a more significant effect on the velocity and the run-out length when the yield strength is low. De Blasio *et al.* [50] give a range of dynamic viscosity *μ*=100–1000 Pa s for large-scale landslide deposits with run-out lengths ranging from 10 to 200 km. Common values used in the numerical simulations of visco-plastic submarine flows do not exceed the value of 300 Pa s and normally range from 30 to 300 Pa s for the simulation of clay and silt-rich sediments [58,61,63]. Considering that the sediment composition in the RBSC is a mixture of clay, silt and sand material [42] a range of 10–300 Pa s was selected for the dynamic viscosity.

Iverson [64] notes that the recorded bulk densities of debris flows rarely fall outside a range of 1800–2300 kg m^−3^. To account for a submerged landslide we use the apparent density of the mixture: *ρ*=(*ρ*_ls_−*ρ*_w_), where *ρ*_ls_ is the bulk landslide density and *ρ*_w_ is the water density (see also [20,65]). For the numerical simulations, we use a reduced density, *ρ*, for the landslide mass where *ρ*_ls_ ranges between 1800 and 2300 kg m^−3^ and *ρ*_w_=1000 kg m^−3^.

Drag can reduce the landslide velocities up to 25% and thus its contribution is significant in the landslide process and tsunami generation [51,62]. As submarine landslides constitute complex processes their maximum velocities are difficult to measure. Fine *et al.* [8] demonstrated that the Grand Banks landslide transformed into a turbidity current that travelled as fast as 17–28 m s^−1^. Numerical simulations of the Storegga Slide show best fit scenarios with maximum velocities below 35 m s^−1^, likely ranging between 25 and 30 m s^−1^ [7]. Simulations of debris flows with the rheology of a Bingham fluid can reach maximum velocities of 70 m s^−1^ [66]. Energetic landslides in the Canary Islands may reach maximum velocities of approximately 150 m s^−1^, however, a large part of the kinetic energy of the landslide evolves above water [12]. In the Hawaiian archipelagos debris flows with peak velocities in the order of 80 m s^−1^ would be required for the long run-outs [5].

**Figure 2.**
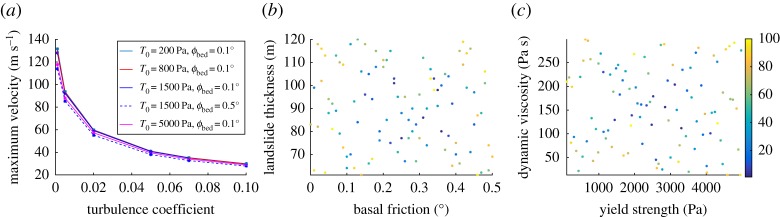
(*a*) Computed maximum velocities for ξ=0.001, 0.0025, 0.02, 0.05, 0.07 and 0.1 and varying yield strength and basal friction (*μ*=10 Pa s and *ρ*=1200 kg m^−3^). (*b*,*c*) Two-dimensional projections of the design points of the parameter values from the LHS design. The colour scale represents individual index of the scenarios (from 1 to 100 scenarios). (Online version in colour.)

A turbulence term leads to reduced maximum velocities and landslide acceleration and can be of great use in the simulations with VolcFlow where the effect of the water drag is not incorporated. In the code, ξ is a non-dimensional coefficient [47] related to the turbulence coefficient initially introduced by Voellmy [52]. The turbulence coefficient has a large impact on the run-out distance and the duration of the landslide. Increased values of ξ can result in decreased run-out lengths and increased duration of the motion. Consequently, some of the input rheological parameters such as the yield strength of the landslide and the basal friction have to be significantly decreased to get a best-fit run-out length. The appropriate value of this coefficient is not known with certainty and the range cannot be easily defined. Some sensitivity analysis tests were implemented to select a range for the turbulence coefficient (figure 2*a*). A range of ξ=0.01–0.08 is selected that roughly corresponds to maximum flow velocities of 30–80 m s^−1^.

The selected intervals of each individual calibration parameter are shown in table 1. In this study, we present the results of 100 numerical experiments computed initially with VolcFlow to simulate the slide and then one-way coupled with VOLNA to model the generated tsunami in §6. To select the input parameters of each scenario the LHS design was used. The advantage of the LHS is that it requires a small number of samples to explore the input space efficiently, contrarily to grids for instance. The space-filling properties of LHS allow for more uniform sampling of the parameters with a smaller number of samples. As a rule of thumb, about 10 values (well sampled) per parameter in a design are sufficient to get a good approximation [67]. In this case, we are using the ‘maximin’ LHS design to select the input parameter values. This space-filling technique maximizes the minimum distance between the points to optimize sampling (figure 2*b*,*c*).

**Table 1.** Input parameter ranges.

parameters	*h*_max_ (m)	*T*_0_ (Pa)	*ϕ*_bed_(^°^)	*μ* (Pa s)	*ρ* (kg m^−3^)	ξ
range	60–120	100–5000	0–0.5	10–300	800–1300	0.01–0.08

## 6. Numerical simulations and posterior distributions

### (a) Simulations set-up for VolcFlow and VOLNA

For the numerical modelling, we combine data from two datasets. The bathymetry data are retrieved from the EMODnet Bathymetry portal and the land elevation data from the GEBCO_08 GRID terrain model. For the landslide modelling, we make use of a digital elevation model (DEM) with dimensions of 200×250 km and spatial resolution Δ*x*=320 m, Δ*y*=500 m. The landslide simulations have a final time of *T*_fin_=5 *h*, with a stopping criterion when the maximum velocity falls beneath the rate of 4 m s^−1^. Below that threshold the toe of the landslide stops advancing and as the velocity decays, motion is constrained in the internal part of the flow. To simulate 5 h of landslide motion a timestep that may vary (Δ*t*=2.5–5 s) to ensure numerical stability is initially used and the results are saved every 5 s. These simulations are used in the calibration against observations of the landslide deposits.

**Figure 3.**
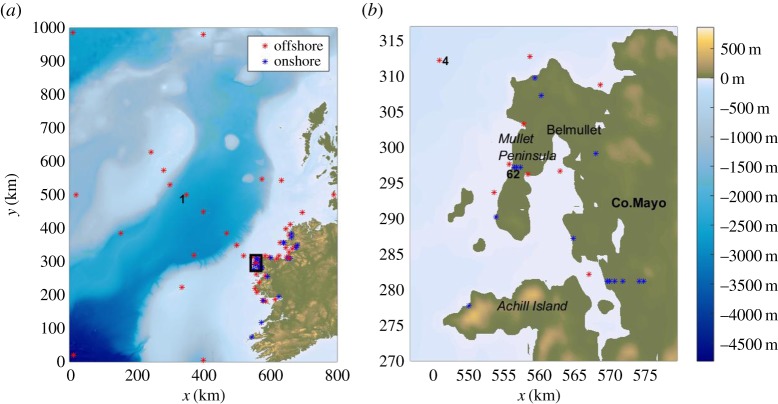
(*a*) The computational domain (source: EMODnet and GEBCO-08) and the virtual gauges used to measure the free surface elevation. The red stars denote the offshore gauges, the blue stars represent onshore gauges. An identifying number is given to each gauge but only the gauges discussed in the text are highlighted here. (*b*) The Mullet Peninsula and the area around Belmullet in Co. Mayo are magnified to better illustrate the wave gauge positions. (Online version in colour.)

To model the complete tsunami life cycle, we use a larger DEM than for the landslide modelling with dimensions of 800×1000 km. The data are smoothed and interpolated onto an unstructured triangular grid. Varying mesh sizes have been tested for mesh convergence leading to an optimal choice of a mesh with a spatial resolution of Δ*x*=Δ*y*=450 m and 5 112 958 nodes. In the tsunami simulations, we incorporate the first hour of the landslide motion as the first moments of sliding are often the most critical for tsunami generation [3]. So, for a refined representation of the motion, in the combined landslide–tsunami modelling, we rerun the numerical simulations in VolcFlow with a timestep of 1 s as inputs to the tsunami model. The tsunami simulations have a duration of 2 h and Δ*t*=0.1 s. To measure the free surface elevation, we introduce 80 gauges in the domain, almost half of which are located offshore and the rest of them onshore, close to the coastline (figure 3). We concentrate the vast majority of the gauges in the area around Belmullet in Co. Mayo (figure 3*b*), as it is expected to be the first and probably most inundated area by the propagating tsunami waves [45].

### (b) Calibration results

**Figure 4.**
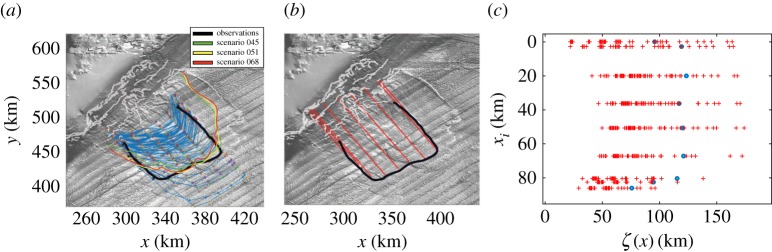
(*a*) Extent of the deposits of the numerical simulations with VolcFlow (blue lines) with respect to the observations of the deposits on the bathymetric data from INSS (black line). The simulated deposits of some of the best fitting scenarios in terms of run-out length are depicted (scenarios 45, 51 and 68, see also table 2). (*b*) Nine lines are considered (indicated with red colour) parallel to the line between the centre of the landslide and the extent of the deposits. The kilometric distance of the simulated deposits is measured along these lines. (*c*) The kilometric distance of the simulated deposits from the points in the slope region (*ζ*(*x*) denotes the distance of the deposits from the slope and *x*_*i*_ denotes the distance between the points on the slope). The blue circles indicate the observations (distance of the field deposits from the slope). (Online version in colour.)

The results of the landslide simulations show that there exists a large variation across the computed run-out lengths of each landslide scenario (figure 4*a*). It is observed that a few of the computed run-out lengths fall roughly in agreement with the length of the deposits on the seabed of the Rockall Trough (e.g. scenarios 45, 51, 68, figure 4*a*, table 2). The southern limit of the deposits is relatively well represented. However, the vast majority of the simulations fail to represent the northern lateral limit of the observed deposits. The simulated deposits that exhibit a substantial run-out length spread much further than the limits of the observed deposition, towards a northeast direction, without exceeding, though, the depositional limits of older episodes of collapse (figure 4*a*).

**Table 2.** Parameters of a few best-fit scenarios.

scenario	*h*_max_ (m)	*T*_0_ (Pa)	*ϕ*_bed_ (^°^)	*μ* (Pa s)	*ρ* (kg m^−3^)	ξ
45	93	794	0.12	133	1069	0.067
51	111	539	0.21	52	1081	0.012
68	95	170	0.26	219	1142	0.039

For the Bayesian calibration, in addition to the selected values *t*_*i*_ of the calibration parameters, the input and output variables $x_{i},Y_{i}^{C},Y_{i}^{F}$ have to be supplied in the model as described in equations (4.1) and (4.2). To do so, we consider the length of the depositional lobes. A set of nine points on the grid, representing the true extent of the field deposits is used (figure 4*b*,*c*). From those points we take nine lines of extent parallel to the bisector of the deposits. The distance of those points from nine fixed points, *x*_*i*_, that fall on the perpendicular with the bisector line on the slope region, represents the response observed in the field, $Y_{i}^{F}$. The distance of the simulated deposits along the same lines of extent corresponds to $Y_{i}^{C}$. We note that the results of the model may be sensitive to a different selection strategy, or different priors for the parameters (here chosen uniform over the selected intervals as there is little scientific evidence that such parameters ought to take particular values within these intervals in the first place). To obtain the posterior distributions of the parameter space, we have specified 2500 MCMC iterations, using an initial burn-in period of 1000 iterations.

**Figure 5.**
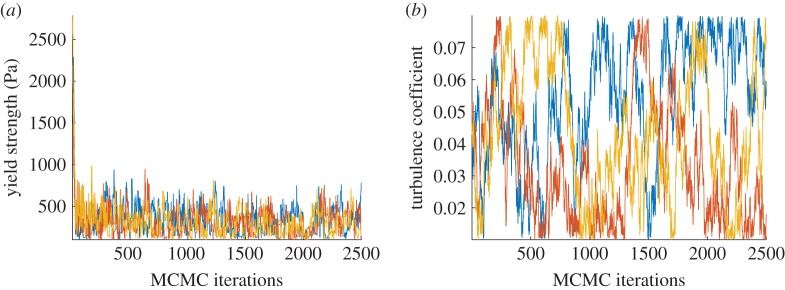
The sample paths of the yield strength (*a*) and the turbulence coefficient (*b*) for 3 chains, 2500 MCMC iterations were performed. (Online version in colour.)

**Figure 6.**
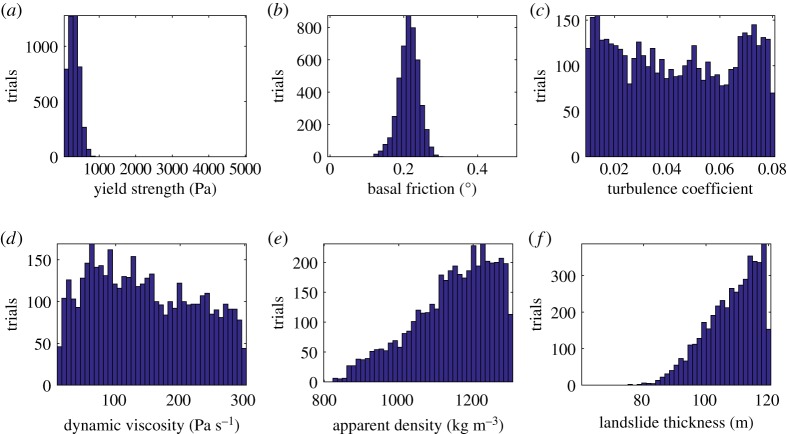
The posterior distributions of the calibration parameters, derived from figure 5. The posterior distributions of the yield strength (*a*), the basal friction (*b*), the turbulence coefficient (*c*), the dynamic viscosity (*d*), the apparent density (*e*) and the landslide thickness (*f*) are displayed. (Online version in colour.)

Figure 5 shows the sample paths for three chains, with the MCMC iterations, corresponding to two out of six calibration parameters. Convergence of the chains is observed mostly for two of the calibration parameters: the yield strength and the basal friction but also to a certain degree for density and thickness. For the rest of the parameters, convergence of the chains is not observed with visual inspection, even when using more chains (10 chains) or running the chains for more iterations (5000 MCMC iterations). The resulting posterior distributions (represented in figure 6, in the form of histograms) allow us to deduce the optimal ranges of the calibration parameters.

The histogram of the yield strength, *T*_0_, is positively skewed and appears to be very constrained, with values located in the lower part of the interval. The posterior distribution shows that the optimal values are lower than 1000 Pa (figure 6*a*). Values above this range do not appear to be optimal for obtaining run-out lengths that match with the observations. The posterior distribution of the basal friction also appears constrained within the interval (figure 6*b*). The shape of the histogram indicates that the optimal values range around the value of 0.2^°^ and within a broader range of nearly 0.1–0.3^°^ (figure 6*b*).

From the posterior distributions of the turbulence coefficient, ξ, and the dynamic viscosity, *μ*, optimal value ranges cannot be drawn with certainty. Indeed, the distributions of the variables exhibit a relatively uniform pattern (figure 6*d*,*e*). The posterior distributions of the apparent density, *ρ*, and the maximum thickness of the landslide, $h_{\max}$, appear to be negatively skewed (figure 6*e*,*f*). The optimal values for both cases are concentrated towards the upper boundary of the intervals (figure 6*f*). The shape of the histograms yield that the optimal values for the density are larger than 1000 kg m^−3^ and for the thickness larger than 90 m.

**Figure 7.**
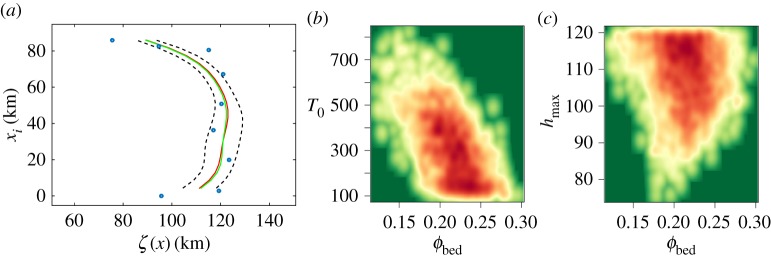
(*a*) The GP prediction of the landslide simulations. The blue circles represent the observations of the landslide's depositional extent on the seabed. The red line denotes the prediction of the landslide simulator. The prediction of the simulator, with the estimated bias added is in green. Credible intervals of 95% are indicated with black dashed lines. (*b*,*c*) Density plot of basal friction with respect to yield strength and maximum slide thickness. The density of the plots accumulates especially between values of *ϕ*_bed_=0.2−0.25^°^. (Online version in colour.)

The predictions of the real process, *ζ*(*x*_*i*_)=*η*(*x*_*i*_,*θ*)+*δ*(*x*_*i*_), are shown in figure 7*a*. The predictions derived only from the emulation of the model are denoted with red colour, the predictions including as well the estimated bias are also displayed (green colour). Furthermore, 95% credible intervals are plotted as black dashed lines. They capture the overall uncertainty arising from the uncertainties in the inputs of the simulations and the various uncertainties in the fit of the emulator. The credible intervals of the predictions are rather wide around some of the observation points. A few observation points, especially the ones located on the lateral extent of the deposits, are not captured by the predictions. The unbiased prediction of the process, which is the sum of the best fit of the model for the optimal values of the input parameter and the bias, appears also to interpolate the observations. Note that there is no clear improvement due to the addition of the bias here. There are two possible explanations. Either there is no bias due to no clear pattern in the difference between the model and the observations after calibration or there is a lack of information that prevents the estimation of the discrepancy. The inclusion of more depositional extents may not help because these are not informative enough due an underlying lack of physical conditions and rather large observation errors.

As we actually infer a joint multivariate distribution of the input parameters (in six dimensions), we now inspect some two-dimensional projections of some combinations of inputs. The density plot of the basal friction is presented as a function of two other calibration parameters: yield strength and landslide thickness (figure 7*b*,*c*). There is an inversely proportional relationship between the basal friction and the yield strength in comparison with the rest of the parameters. It appears that to simulate the observed length of the landslide deposits, the yield strength must decrease when the basal friction increases and vice versa. Indeed, this physically makes sense as the two parameters influence the resulting landslide extent in a similar way. Regarding the thickness of the landslide, the density of the plot accumulates in the higher boundary of the thickness interval and particularly in values between 110 and 120 m. The density plots of the remaining calibration parameters (ξ, *μ* and *ρ*) are also constrained by the optimal range of *ϕ*_bed_.

### (c) Tsunami simulations

**Figure 8.**
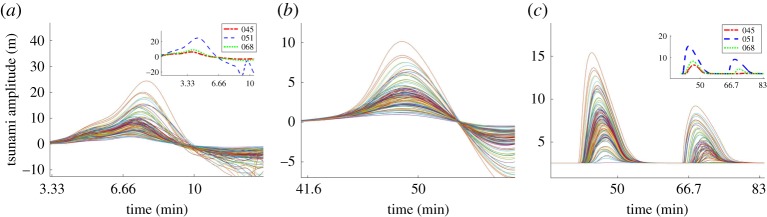
(*a*–*c*) The maximum free surface elevation resulting from 100 simulations with a real time duration of 2 h. Gauge 1 is located close to the source (*x*,*y*=(350,500 km)). Gauge 4 (*x*,*y*=(545.8,312.2 km)) is a few kilometres away from the shoreline. Gauge 62 (*x*, *y*=(556.4,297.1 km)) is located on land above the sea level (see also figure 3). Also shown inside panels (*a*,*c*) are simulation numbers 45, 51 and 68, over the same time intervals, respectively (as large variations of gauges occur at different times). (Online version in colour.)

The results of the numerical simulations of the generated tsunamis, using the uncalibrated set of 100 runs from the LHS, show a large variability in the amplitude of the waves (figure 8). When the tsunamis start propagating away from the source, the wave forms tend to follow a similar pattern and the wave peak arrival times seem to converge (gauges 04; 62). This is explained by the dependency of the tsunami wave speed on the depth of the basin, which remains constant during the simulations. The wave gauges located close to Belmullet are the first onshore gauges to measure the free surface elevation, approximately 50 minutes after the tsunami generation (figure 8*c*). The maximum water surface elevation varies over a range of 2.7 to 24.4 m at gauge 1, whereas the tsunami run-up at gauge 62 ranges between 0 and 15.4 m. The landslide scenario resulting in the maximum recorded tsunami amplitudes is scenario 51. The wave amplitudes resulting from landslide scenarios 45, 51 and 68, which are the ones closer to the observed deposition, are also shown (figure 8).

As VOLNA is an NSWE solver, frequency dispersion of landslide tsunamis is not accounted for. The impact of frequency dispersion is typically visible on landslide tsunami propagation [11,12,22,68]. Dispersive effects often influence the form of the wave train and the coastal run-up [14,22,24,27,68,]. During generation, the length-to-depth ratio of the landslide influences the wave components of the tsunamis [14]. Dispersive effects are enhanced by the shorter wave components of landslide tsunamis and the frequently dipole shape of the source which leads to radial spreading of the tsunamis during propagation [14,22,69,70].

Computations of landslide tsunamis at the Ormen Lange/Storegga area made with a non-dispersive code show good correlation between the maximum free-surface elevations, and the product of the landside volume with the initial acceleration, or the product of the volume with the maximum velocity [9]. On the other hand, another case study in the same area shows that for a landslide of smaller volume the dispersive effects are much larger. The results show a 25% difference in the maximum surface elevation which decreases immediately after the landslide has stopped [9,71].

The effect of dispersion is also influenced by main landslide characteristics such as the landslide acceleration and velocity, and the landslide length [71]. Dispersive effects become more important with increasing landslide acceleration and velocity; on the other hand, dispersive effects become less important with increasing landslide lengths [71]. For long landslides (as opposed to short, impulsive events), the time evolution of the landslide is controlled by the landslide acceleration which in sequence influences the tsunami generation and the degree of dispersion [14]. During large, strongly subcritical motion the dispersive effects can become minor [71]. Such an example is the Storegga slide where the slide characteristics may render the effect of dispersion insignificant for distances much larger than 200 km away from the source [7,22,71].

Given the variety of conclusions on dispersion, Glimsdal *et al.* [22] have introduced a dispersive parameter, *τ*, to assess the importance of dispersion on the tsunami wave characteristics. The dispersive parameter can be used as a tool for indication rather than an exact calculation. It is given by

6.1 $$\tau =\frac{6z_{o}^{2}L}{\lambda^{3}}$$

where *z*_*o*_ is the equilibrium depth, *L* the distance from the source and λ the wavelength.

For earthquake tsunamis, the source width (described as the width of the fault when the point of observation is lateral to the fault) can be used to characterize the importance of dispersive effects on the waves. For landslide tsunamis, the landslide transformation during motion and uncertainties linked with the nature of the failure, complicate the process. Thus, λ, in that case can be derived from the newly generated wave as twice the distance between the first crest of the wave and the point in the front of the wave where the elevation is at 10% of the crest's height [22]. The strong influence of the source width/initial wavelength on the value of *τ* and thus on the dispersive effects becomes clear from equation (6.1). Based on observations from different cases, Glimsdal *et al.* [22] have concluded that for *τ*<0.01 the effect of dispersion is small but it becomes non-negligible when *τ* is greater than 0.1.

**Figure 9.**
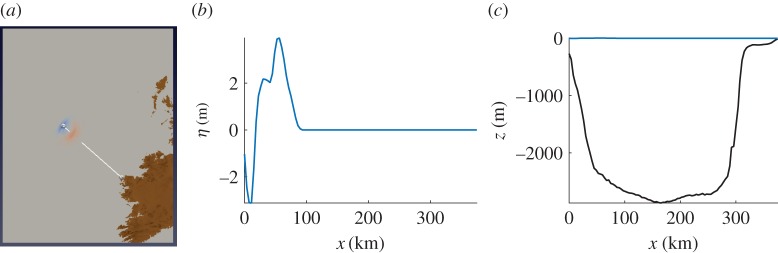
(*a*) The computational domain at *t*=240 s, the white line indicates the distance between the source region and the first point of inundation on the coast. (*b*) Transect of the free surface elevation. (*c*) Transect of the bathymetry (black) and the free surface elevation (blue). (Online version in colour.)

The estimated volume of collapse in the RBSC is 400 km^3^, the width of the failure area in the slope region is approximately 70–80 km [42]. The collapse can be considered of large dimensions and we thus attempt to provide a rough estimation of the dispersive parameter as described in equation (6.1). To do so, we take a transect from the source region up to the first point of inundation in the Mullet peninsula (figure 9*a*). This distance is approximately 350 km and the average depth between the two points is found *z*_*o*_≈−2 km (figure 9*c*). Following [22], we consider scenario 68 at *t*=240 s, the wavelength is estimated ∼60 km (figure 9*b*). At a distance of *L*=100 km from the source region, the dispersive parameter becomes 0.01 and for *L*=350 km *τ* becomes 0.039. These values reveal that the effect of dispersion in the beginning of the wave generation is small and becomes moderate as the waves propagate towards the shoreline.

The primary aim of this work focuses on the methodology that can be used to account for uncertainty quantification and sensitivity analysis on a real case study. As a consequence dispersive effects were not further investigated. However, comparative studies will be the focus of future work to accurately assess the geophysical aspects of landslide tsunamis in the region.

## 7. Statistical emulation of the coupled process

One of the advantages of using a statistical emulator as a substitute for a deterministic model is the ability to access in a faster and less computationally expensive way the results of otherwise costly numerical simulators. Although a statistical surrogate should not be treated as a complete replacement of the simulator, it has an indisputable value for uncertainty quantification purposes. In this study, we emulate the coupled process of submarine sliding and tsunami generation in the RBSC. We utilize known data from computer experiments to build a GP that can be then used as a surrogate of the computational process. Kriging methods are used in geostatistics for interpolation and used here to predict the values of the GP outputs as well as corresponding uncertainties, with many implementations (here [72]). The principal concept of kriging is to use the weighted average of the neighbouring points (in space for geostatistics, or here in the input space of the computer model) in order to estimate the value at any desired point in the input space. The set of the responses corresponds to the maximum tsunami amplitude computed for a selected wave gauge: we carry out this study gauge by gauge. Our main objective is to use the emulator in order to make predictions of the maximum water surface elevations at a specific wave gauge for any set of unknown parameter values that fall within the selected range.

Consider a set of m design sites in the input space *x*=[*x*_1_…*x*_*m*_]^*T*^ and *y*=[*y*_1_…*y*_*m*_]^*T*^ responses with *x*_*i*_∈*R*^*n*^, *y*_*i*_∈*R*^*q*^. Using the design points in the input space and the corresponding response, a GP is tuned to mimic the input–output relationship and the kriging model is able to calculate predictions anywhere in the input space using the GP structure. Both the mean function (which is in general a regression onto other functions) and the correlation function have to be set to fix the GP. However, the mean is often set to zero when there is not enough evidence of a link to other functions; in that case the correlation satisfactorily captures the variability. The key objective is, based on the provided information, to build a GP approximation model which can be later used to predict the physical process using kriging. We give a brief description of the approach. Given a *n*-dimensional input *x*, subset of *R*^*n*^, the deterministic response *y*(*x*)∈*R* can be written as

7.1 y(x)=F(β,x)+z(x),

where *F*(*β*,*x*)=[*f*(*x*)]^*T*^*β*,*f*(*x*)=[*f*_1_(*x*)…*f*_*p*_(*x*)] is the regression model. The approximation error is denoted by *z*. The approximation error is assumed to behave as a GP in the region of interest; its probability distribution has zero mean and finite variance with a particular covariance structure. The approximation model of the fitted response $\hat{y}(x)$ can be described as a function of the regression model *F* and a fitted random process $\hat{z}$ that models *z*

7.2 $$\hat{y}(x)=F(\hat{\beta },x)+\hat{z}(x).$$

A mean function of *F*=0 is chosen here: no regression term *F* is present. Indeed, we found that trying to include such terms was not beneficial here; note that it could be beneficial in other settings [31]. The covariance function between two *n*-dimensional trial sites *x* and *x*′ is given by

7.3 $$E[z(x)z(x^{\prime })]=\sigma^{2}R(\vartheta ,x,x^{\prime }),$$

where *σ*^2^ is the process variance and *R*(*ϑ*,*x*,*x*′) is the correlation model of the fitted GP, which is a function of the correlation function parameters *ϑ*.

For the set of *x*=[*x*_1_…*x*_*m*_]^*T*^ design sites and *Y* responses, the vector of correlations between inputs and the predicted location *x* becomes

7.4 $$r(x)={\left[R(\vartheta ,x_{1},x)\ldots R(\vartheta ,x_{m},x)\right]}^{T},$$

whereas the matrix of correlations across the input space, using the initial design, is simply

7.5 $$R=\left[R(\vartheta ,x_{i},x_{j})\right],\;i,j=1,\ldots ,m.$$

To build the model and make predictions for our case, we use a set of m input parameters corresponding to m=100 scenarios and *Y*_m_ responses. The set of the inputs consists of the values of the six parameters $(h_{\max},T_{0},\phi_{\mathrm{bed}},\xi ,\mu$ and *ρ*) used in the simulation of the numerical experiments and the Bayesian calibration. To build the predictor, a correlation model and the appropriate choice of correlation function parameters *ϑ* are required. We select a Gaussian correlation model, $R(\vartheta ,x,x^{\prime })=\prod_{i=1}^{n}\exp(-\vartheta_{i}{\left(x_{i}-x_{i}^{\prime }\right)}^{2}),$ where *i* runs across six dimensions of inputs. The model was chosen for its flexibility, broad generality, and evidence of a satisfactory behaviour in various contexts. Our preliminary analyses showed little influence of these choices here. In our case with no regression function, the kriging predictor is simply given by

7.6 $$\hat{y}(x)=r{\left(x\right)}^{T}R^{-1}Y.$$

The mean squared error (MSE) of the kriging predictor can be also computed as

7.7 $$\mathrm{MSE}=E[(\hat{y}(x)-y{\left(x)\right)}^{2}]=\sigma^{2}(1-r^{T}R^{-1}r).$$

For the selection of the optimal values of the correlation parameters, the lower and upper boundaries of *ϑ* are selected to be 0.001 and 10 (as all parameters are normalized). We use a maximum-likelihood (ML) approach to estimate *ϑ*. As numerical maximization can be challenging, we carry out this maximization for several initial starting points. To homogeneously draw the initial starting points for *ϑ*, we use a small LHS to create a different set of 20 initial scenarios. The comparisons show that there is some variability in the absolute difference between the predictions and the true response influenced by these starting points for *ϑ* if we had chosen these starting points as guesses.

Hence, we run ML to obtain a final estimate for ϑ* repeatedly picking the 20 starting points *ϑ*, in order to allow the maximization under an exploration–exploitation paradigm. After a limited number of runs, the final estimate ϑ* converges towards a certain value for all starting points (most of them yield a similar value). To evaluate each of these final estimates, we do cross-validation tests with the implementation of LOO diagnostics. The governing principle in LOO is to estimate ϑ* each time neglecting one out of 100 design sites and compare the stochastic predictions with the true deterministic response of the process. We compute our LOO diagnostics by comparing the absolute difference between the predictions and the true process. We select the value of ϑ* that results in the best LOO prediction (smaller absolute difference between stochastic predictions and deterministic response for a fixed set of inputs) among our 20 possible values. We eventually pick *ϑ*=[0.0321,0.0139,1.2427,0.001,0.001,0.1498].

**Figure 10.**
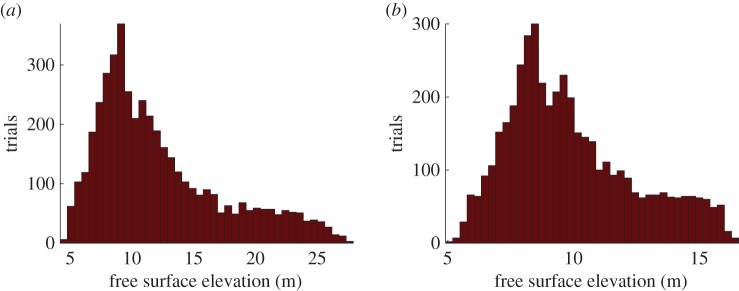
(*a*,*b*) Distributions of the emulated (fast surrogate predictions) maximum free surface elevation in gauges 01 and 62 (gauge locations shown in figure 3*b*). Emulated predictions for *n* = 4500 scenarios whose distributions are specified from the calibration of the landslide simulations against observations of deposits. (Online version in colour.)

By setting all the above parameters, the emulator is built and can be therefore used for predictions. To quantify the uncertainty governing the tsunami amplitudes generated by the examined landslide example in the RBSC, we make use of the results of the Bayesian calibration. Taking as input the values of the calibrated parameters from the posterior distributions (figure 6), we make predictions for 4500 scenarios. The predictions take a very small amount of time (*t*≈1 s) to yield 4500 expected maximum tsunami elevations at each desired wave gauge. The results constitute a probabilistic statement about the maximum water surface elevation at this specific location. The distributions of the predictions are presented in the form of histograms for the offshore gauge 01 and the onshore gauge 62 (figure 10, see also figure 3).

The histograms of the maximum free surface elevation in gauges 01 and 62 exhibit a positively skewed probabilistic distribution. At gauge 01, the majority of the predictions give maximum tsunami amplitudes ranging between 7 and 12 m (figure 10*a*). The highest predicted tsunami amplitudes may exceed 15 m in more infrequent cases. At gauge 62, the most probable maximum run-up is approximately 8 m. The majority of the predicted maximum run-up heights range between 7 and 10 m and they rarely exceed a value of 16 m (figure 10*b*). Following the same process, predictions and assumptions for the rest of the gauges can be made with the aid of the statistical emulator.

**Figure 11.**
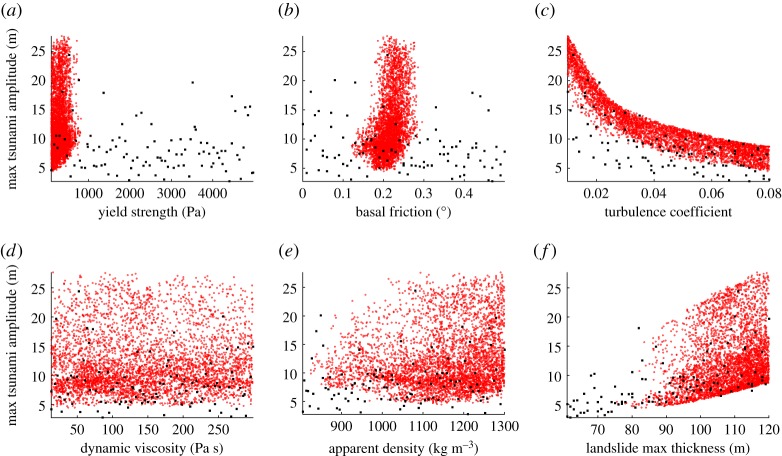
(*a*–*f*) Scatter plot projections of the maximum tsunami amplitudes at gauge 01 with respect to the increase or decrease in the value of each individual calibration parameter. The black squares represent the results of the 100 numerical simulations at gauge 01. The red dots denote the results of the 4500 emulated predictions at the same gauge. (Online version in colour.)

The emulation allows some further exploration of the sensitivity of the maximum tsunami amplitudes to the varying input parameters due to the possibility of running the model at many more locations in the input space. Gauge 01 is selected to illustrate the sensitivity analysis (figure 11). The maximum tsunami amplitude variations with respect to variations in the values of the calibration parameters are plotted, deriving from the 100 numerical simulations, the emulation, and the 4500 predictions. Note that in comparison with the initial 100 numerical simulations, the kriging predictions are constrained within the optimal ranges of the parameters as seen clearly in figure 11*a*,*b*.

The plots show an almost linear relationship between the maximum free surface elevation and the maximum landslide thickness (figure 11*f*). However, the relationship between the maximum free surface elevation and the turbulence coefficient appears to be slightly nonlinear (figure 11*c*). We note that the tsunami amplitude increases when $h_{\max}$ increases and ξ decreases. It is likely that a similar pattern would be observed for the added mass coefficient as it also contributes to the kinetic energy of the landslide. These results are in agreement with the up-to-date scientific knowledge that the features of landslide tsunamis depend, among other, on the thickness and the kinetic energy of the landslide [1]. The rest of the calibration parameters appear to be much less influential.

## 8. Conclusion

In this study, we implemented a Bayesian calibration to explore the optimal values of the landslide parameters used in the numerical modelling of submarine sliding and tsunami generation in the RBSC. A comprehensive statistical emulation of the one-way coupled numerical process was then employed to explore the probabilistic maximum tsunami elevation at specific locations. The possible tsunami amplitudes generated by a submarine slope collapse in the lower slope region of the Rockall Bank were estimated with the use of the emulator.

The posterior distributions of the parameters, resulting from the calibration, show that the yield strength and the basal friction of the landslide can affect critically the run-out length of the flow. The optimal values of both parameters appear to be very constrained within the initially attributed range (*T*_0_<1000 Pa and *ϕ*_bed_∼0.2^°^) in order to match the observed run-out. The density plots show an inversely proportional relationship between the yield strength and the basal friction. The calibrated maximum slide thickness is likely to be within the higher values of the initial range: *h*_max_>100 m are likely to help match modelled landslides to the desired run-out length. The apparent density is also negatively skewed with optimal values higher than 1000 kg m^−3^. The posterior distributions of the turbulence coefficient and the dynamic viscosity show a relatively uniform distribution. No significant effect of the two parameters on the simulated run-out of the flow can be deduced from the calibration.

Within the limitations of our modelling assumptions, we employed the emulator to make predictions of the maximum tsunami amplitudes using the posterior distributions of the calibration parameters. In total, predictions of 4500 cases were made in only a few moments of time (*t*≈1 s). The time of the predictions is excessively fast when compared to the computational speed of one run of the combined models (roughly 11–13 h for each VOLNA run with 2 GPUs). The probabilistic distributions of the predicted maximum tsunami amplitudes were presented for two locations: an offshore gauge close to the generation region (gauge 01) and an onshore gauge in the Mullet Peninsula (gauge 62). The predictions at gauge 01 show that the tsunami amplitudes of a similar landslide would more likely vary between 7 and 12 m. Likewise, at gauge 62 the predictions show that although cases of higher tsunami run-up (>10 m) are not excluded, they exhibit a less common occurrence rate in comparison to lower run-up heights (between 7 and 10 m). Similar predictions can be made for the rest of the gauges.

Thanks to the large number of fast and accurate predictions the emulator can be used to perform sensitivity tests. A sensitivity analysis of the maximum water surface elevation with respect to the varying parameters shows that variations in the simulated and predicted tsunami amplitudes are strongly linked to two calibration parameters: the landslide thickness and the turbulence coefficient. In more detail, the tsunami amplitude increases when the landslide thickness increases and the turbulence coefficient, hence the maximum velocity and possibly initial acceleration of the landslide, decreases. The rest of the calibration parameters (yield strength, basal friction, dynamic viscosity and apparent density) do not appear to have a significant effect on the magnitude of the tsunami amplitudes. As so, to achieve the optimal run-out length of the flow the yield strength and the basal friction are critical whereas the turbulence coefficient does not hold such an important role. Different rheological parameters can thus affect the magnitude of different processes and their influence should not be disregarded.

We note that building a statistical surrogate of a deterministic code is not a replacement for scientifically sound simulations of a physical process in the first place. It forms a verified, fast and inexpensive technique to make predictions of the process and quantify uncertainty, particularly in cases where a large number of simulations has to be considered and it is difficult or computationally expensive to run the codes. In most instances, the results are only applicable for the specific case examined. A diverse parameter selection, or an alternative scientific approach to the problem, will possibly generate different results and another calibration and emulation should be performed on a case-by-case situation.

To perform the calibration and the emulation, we used data from the RBSC which were available to us during the process. As scientific research advances day by day with technological progress and the results of new studies, new information for the event can be revealed. Ongoing research in the subject shows that the deposits of the most recent event have a slightly different shape than the one we used in this study [42]. As the main objective was to focus on the statistical process used for the calibration and emulation of a real case, such data are not yet incorporated. A future research topic would be to incorporate these uncertainties; there is scope for deepening our investigation to include the latest updates.

This work forms one of the first attempts to study numerically slope failure in the RBSC, and built a statistical emulator of a real case study for uncertainty quantification and sensitivity analysis. Driven by this, future research endeavours may focus on different approaches to study parametric variability. In addition, to adequately account for the physics of the phenomenon, a comparison and verification of the modelling strategy employed here with the results of fully coupled modelling of the event, including incorporation of the landslide tsunami dispersive features, should also be considered. The construction of extended hazard maps for the area could also be useful for future hazard assessments. To do so, a slope stability assessment and a different meshing strategy in the modelling, with mesh refinement on the coastal areas, would be of critical importance. Overall, the approach followed in this study forms a tool to study a certain parametric variability to understand the uncertainty governing submarine sliding and tsunami generation in the RBSC. It can be used as the basis for future probabilistic assessments of landslide-generated tsunamis in other regions of the world prone to such events.

## Data accessibility

The bathymetry data derived from the EMODnet Bathymetry portal: http://www.emodnet.eu/bathymetry. The land elevation data derived from the GEBCO portal: http://www.gebco.net/data_and_products/gridded_bathymetry_data/version_20100927/.

## References

[1] Ward SN 2001 Landslide tsunami. *J. Geophys. Res. Solid Earth* 106, 11 201–11 215. (doi:10.1029/2000JB900450)

[2] Bardet JP, Synolakis EC, Davies LH, Imamura F, Okal AE 2003 Landslide tsunamis: recent findings and research directions. *Pure Appl. Geophys.* 160, 1793–1809. (doi:10.1007/s00024-003-2406-0)

[3] Harbitz CB, Løvholt F, Pedersen G, Masson DG 2006 Mechanisms of tsunami generation by submarine landslides: a short review. *Norwegian J. Geol.* 86, 255–264.

[4] Hühnerbach V, Masson D 2004 Landslides in the North Atlantic and its adjacent seas: an analysis of their morphology, setting and behaviour. *Mar. Geol.* 213, 343–362. (doi:10.1016/j.margeo.2004.10.013)

[5] Masson DG, Harbitz CB, Wynn RB, Pedersen G, Løvholt F 2006 Submarine landslides: processes, triggers and hazard prediction. *Phil. Trans. R. Soc. A* 364, 2009–2039. (doi:10.1098/rsta.2006.1810) 16844646 10.1098/rsta.2006.1810

[6] Ward SN, Day S 2001 Cumbre Vieja Volcano-Potential collapse and tsunami at La Palma, Canary Islands. *Geophys. Res. Lett.* 28, 3397–3400. (doi:10.1029/2001GL013110)

[7] Bondevik S, Løvholt F, Harbitz C, Mangerud J, Dawson A, Svendsen JI 2005 The Storegga Slide tsunami—comparing field observations with numerical simulations. *Mar. Petroleum Geol.* 22, 195–208. (doi:10.1016/j.marpetgeo.2004.10.003)

[8] Fine I, Rabinovich A, Bornhold B, Thomson R, Kulikov E 2005 The Grand Banks landslide-generated tsunami of November 18, 1929: preliminary analysis and numerical modeling. *Mar. Geol.* 215, 45–57. (doi:10.1016/j.margeo.2004.11.007)

[9] Løvholt F, Harbitz CB, Haugen KB 2005 A parametric study of tsunamis generated by submarine slides in the Ormen Lange/Storegga area off western Norway. *Mar. Petroleum Geol.* 22, 219–231. (doi:10.1016/j.marpetgeo.2004.10.017)

[10] Geist EL, Lynett PJ, Chaytor JD 2009 Hydrodynamic modeling of tsunamis from the Currituck landslide. *Mar. Geol.* 264, 41–52. (doi:10.1016/j.margeo.2008.09.005)

[11] Løvholt F, Pedersen G, Gisler G 2008 Oceanic propagation of a potential tsunami from the La Palma Island. *J. Geophys. Res.* 113, C09026 (doi:10.1029/2007JC004603)

[12] Abadie SM, Harris JC, Grilli ST, Fabre R 2012 Numerical modeling of tsunami waves generated by the flank collapse of the Cumbre Vieja Volcano (La Palma, Canary Islands): tsunami source and near field effects: modeling of La Palma Tsunami. *J. Geophys. Res. Oceans* 117, C05030 (doi:10.1029/2011JC007646)

[13] Grilli ST *et al.* 2015 Modeling of SMF tsunami hazard along the upper US East Coast: detailed impact around Ocean City, MD. *Nat. Hazards* 76, 705–746. (doi:10.1007/s11069-014-1522-8)

[14] Løvholt F, Pedersen G, Harbitz CB, Glimsdal S, Kim J 2015 On the characteristics of landslide tsunamis. *Phil. Trans. R. Soc. A* 373, 20140376 (doi:10.1098/rsta.2014.0376) 26392615 10.1098/rsta.2014.0376 PMC4608034

[15] Yavari-Ramshe S, Ataie-Ashtiani B 2016 Numerical modeling of subaerial and submarine landslide-generated tsunami waves-recent advances and future challenges. *Landslides* 13, 1325–1368. (doi:10.1007/s10346-016-0734-2)

[16] Synolakis CE, Bardet JP, Borrero JC, Davies HL, Okal EA, Silver EA, Sweet S, Tappin DR 2002 The slump origin of the 1998 Papua New Guinea Tsunami. *Proc. R. Soc. Lond. A* 458, 763–789. (doi:10.1098/rspa.2001.0915)

[17] Harbitz C, Glimsdal S, Løvholt F, Kveldsvik V, Pedersen G, Jensen A 2014 Rockslide tsunamis in complex fjords: from an unstable rock slope at Åkerneset to tsunami risk in western Norway. *Coastal Eng.* 88, 101–122. (doi:10.1016/j.coastaleng.2014.02.003)

[18] Ulvrova M, Paris R, Nomikou P, Kelfoun K, Leibrandt S, Tappin D, McCoy F 2016 Source of the tsunami generated by the 1650 AD eruption of Kolumbo submarine volcano (Aegean Sea, Greece). *J. Volcanol. Geothermal Res.* 321, 125–139. (doi:10.1016/j.jvolgeores.2016.04.034)

[19] Lynett PJ, Borrero JC, Liu PLF, Synolakis CE 2003 Field survey and numerical simulations: a review of the 1998 Papua New Guinea tsunami. *Pure Appl. Geophys.* 160, 2119–2146. (doi:10.1007/s00024-003-2422-0)

[20] Kelfoun K, Giachetti T, Labazuy P 2010 Landslide-generated tsunamis at Réunion Island. *J. Geophys. Res.* 115, F04012 (doi:10.1029/2009JF001381)

[21] Labbé M, Donnadieu C, Daubord C, Hébert H 2012 Refined numerical modeling of the 1979 tsunami in Nice (French Riviera): comparison with coastal data: Modeling of the 1979 Nice tsunami. *J. Geophys. Res. Earth Surf.* 117, F01008.

[22] Glimsdal S, Pedersen GK, Harbitz CB, Løvholt F 2013 Dispersion of tsunamis: does it really matter? *Nat. Hazards Earth Syst. Sci.* 13, 1507–1526. (doi:10.5194/nhess-13-1507-2013)

[23] Lynett P, Liu PL-F 2005 A numerical study of the run-up generated by three-dimensional landslides. *J. Geophys. Res.* 110, C03006 (doi:10.1029/2004JC002443)

[24] Kirby JT, Shi F, Tehranirad B, Harris JC, Grilli ST 2013 Dispersive tsunami waves in the ocean: model equations and sensitivity to dispersion and Coriolis effects. *Ocean Modell.* 62, 39–55. (doi:10.1016/j.ocemod.2012.11.009)

[25] Abadie S, Morichon D, Grilli S, Glockner S 2008 VOF/Navier-Stokes numerical modeling of surface waves generated by subaerial landslides. *La Houille Blanche* 1, 21–26. (doi:10.1051/lhb:2008001)

[26] Davies DR, Wilson CR, Kramer SC 2011 Fluidity: a fully unstructured anisotropic adaptive mesh computational modeling framework for geodynamics: Fluidity-modeling geodynamical flows. *Geochem. Geophys. Geosyst.* 12, Q06001 (doi:10.1029/2011GC003551)

[27] Ma G, Shi F, Kirby JT 2012 Shock-capturing non-hydrostatic model for fully dispersive surface wave processes. *Ocean Modell.* 43–44, 22–35. (doi:10.1016/j.ocemod.2011.12.002)

[28] Tappin DR *et al.* 2014 Did a submarine landslide contribute to the 2011 Tohoku tsunami? *Mar. Geol.* 357, 344–361. (doi:10.1016/j.margeo.2014.09.043)

[29] Tehranirad B, Harris JC, Grilli AR, Grilli ST, Abadie S, Kirby JT, Shi F 2015 Far-field tsunami impact in the North Atlantic basin from large scale flank collapses of the Cumbre Vieja Volcano, La Palma. *Pure Appl. Geophys.* 172, 3589–3616. (doi:10.1007/s00024-015-1135-5)

[30] Behrens J, Dias F 2015 New computational methods in tsunami science. *Phil. Trans. R. Soc. A* 373, 20140382 (doi:10.1098/rsta.2014.0382) 26392612 10.1098/rsta.2014.0382

[31] Sarri A, Guillas S, Dias F 2012 Statistical emulation of a tsunami model for sensitivity analysis and uncertainty quantification. *Nat. Hazards Earth Syst. Sci.* 12, 2003–2018. (doi:10.5194/nhess-12-2003-2012)

[32] Sraj I, Mandli KT, Knio OM, Dawson CN, Hoteit I 2014 Uncertainty quantification and inference of Manning‘s friction coefficients using DART buoy data during the Tohoku tsunami. *Ocean Modell.* 83, 82–97. (doi:10.1016/j.ocemod.2014.09.001)

[33] Beck J, Guillas S 2016 Sequential design with mutual information for computer experiments (MICE): emulation of a tsunami model. *SIAM/ASA J. Uncertainty Quantification* 4, 739–766. (doi:10.1137/140989613)

[34] Sammarco P, Renzi E 2008 Landslide tsunamis propagating along a plane beach. *J. Fluid Mech.* 598, 107–119. (doi:10.1017/S0022112007009731)

[35] Georgiopoulou A, Shannon PM, Sacchetti F, Haughton PD, Benetti S 2013 Basement-controlled multiple slope collapses, Rockall Bank slide complex, NE Atlantic. *Mar. Geol.* 336, 198–214. (doi:10.1016/j.margeo.2012.12.003)

[36] Kelfoun K, Druitt TH 2005 Numerical modeling of the emplacement of Socompa rock avalanche, Chile. *J. Geophys. Res.* 110, B12202 (doi:10.1029/2005JB003758)

[37] Dutykh D, Poncet R, Dias F 2011 The VOLNA code for the numerical modeling of tsunami waves: generation, propagation and inundation. *Eur. J. Mech. B/Fluids* 30, 598–615. (doi:10.1016/j.euromechflu.2011.05.005)

[38] Kennedy MC, O’Hagan A 2001 Bayesian calibration of computer models. *J. R. Stat. Soc. Ser. B* 63, 425–464. (doi:10.1111/1467-9868.00294)

[39] Guillas S, Glover N, Malki-Epshtein L 2014 Bayesian calibration of the constants of the *k*−*ε* turbulence model for a CFD model of street canyon flow. *Comp. Methods Appl. Mech. Eng.* 279, 536–553. (doi:10.1016/j.cma.2014.06.008)

[40] Chib S, Greenberg E 1995 Understanding the Metropolis–Hastings algorithm. *Am. Stat.* 49, 327–335. (doi:10.1080/00031305.1995.10476177)

[41] Elliott GM, Shannon PM, Haughton PD, Ovrebo LK 2010 The Rockall Bank mass flow: collapse of a moated contourite drift onlapping the eastern flank of Rockall Bank, west of Ireland. *Mar. Petrol. Geol.* 27, 92–107. (doi:10.1016/j.marpetgeo.2009.07.006)

[42] Georgiopoulou A, Krastel S, Trappe K, Shannon PM, Haughton P, McCarron S In preparation. Distinguishing individual failure events in the Rockall Bank Slide Complex, NorthEast Atlantic.

[43] Georgiopoulou A, Krastel S, Cruise participants. 2012 RV Celtic Explorer CE11011, 7–21 September 2011 (Hamburg to Galway): seismic investigation of the geology and oceanography of the Northern Rockall Trough, NE Atlantic. Cruise report, pp. 56. Oranmore, Ireland: marine Institute.

[44] Clark CD, Hughes ALC, Greenwood SL, Jordan C, Sejrup HP 2012 Pattern and timing of retreat of the last British-Irish Ice Sheet. *Quat. Sci. Rev.* 44, 112–146. (doi:10.1016/j.quascirev.2010.07.019)

[45] Salmanidou DM, Georgiopoulou A, Guillas S, Dias F 2015 Numerical modelling of mass failure processes and tsunamigenesis on the Rockall Trough, NE Atlantic Ocean. In *Proc. 25th Int. Offshore and Polar Engineering Conf., Kona, HI, 21–26 June*. 10.1098/rspa.2017.0026 PMC5415699 28484339

[46] Kelfoun K, Samaniego P, Palacios P, Barba D 2009 Testing the suitability of frictional behaviour for pyroclastic flow simulation by comparison with a well-constrained eruption at Tungurahua volcano (Ecuador). *Bull. Volcanol.* 71, 1057–1075. (doi:10.1007/s00445-009-0286-6)

[47] Kelfoun K 2011 Suitability of simple rheological laws for the numerical simulation of dense pyroclastic flows and long-runout volcanic avalanches. *J. Geophys. Res.* 116, B08209 (doi:10.1029/2010JB007622)

[48] Huang X, García MH 1997 A perturbation solution for Bingham-Plastic mudflows. *J. Hydraulic Eng.* 123, 986–994. (doi:10.1061/(ASCE)0733-9429(1997)123:11(986))

[49] Imran J, Parker G, Locat J, Lee H 2001 1D numerical model of muddy subaqueous and subaerial debris flows. *J. Hydraulic Eng.* 127, 959–968. (doi:10.1061/(ASCE)0733-9429(2001)127:11(959))

[50] De Blasio FV, Engvik L, Harbitz CB, Elverhøi A 2004 Hydroplaning and submarine debris flows. *J. Geophys. Res. Oceans* 109, C01002 (doi:10.1029/2002JC001714)

[51] Norem H, Locat J, Schieldrop B 1990 An approach to the physics and the modeling of submarine flowslides. *Mar. Geotechnol.* 9, 93–111. (doi:10.1080/10641199009388233)

[52] Voellmy A 1955 Über die zerstörungskraft von Lawinen. *Schweizerische Bauzeitung* 73, 212–217.

[53] Watts P 2000 Tsunami features of solid block underwater landslides. *J. Waterway Port Coastal Ocean Eng.* 126, 144–152. (doi:10.1061/(ASCE)0733-950X(2000)126:3(144))

[54] Dias F, Dutykh D, O’ Brien L, Renzi E, Stefanakis T 2014 On the modelling of tsunami generation and tsunami inundation. *Proc. IUTAM* 10, 338–355. (doi:10.1016/j.piutam.2014.01.029)

[55] Stefanakis TS, Contal E, Vayatis N, Dias F, Synolakis CE 2014 Can small islands protect nearby coasts from tsunamis? An active experimental design approach. *Proc. R. Soc. A* 470, 20140575 (doi:10.1098/rspa.2014.0575)

[56] Tuo R, Wu CFJ 2015 Efficient calibration for imperfect computer models. *Ann. Stat.* 43, 2331–2352. (doi:10.1214/15-AOS1314)

[57] Plumlee M 2016 Bayesian calibration of inexact computer models. *J. Am. Stat. Assoc*. (doi:10.1080/01621459.2016.1211016)

[58] Marr JG, Elverhøi A, Harbitz C, Imran J, Harff P 2002 Numerical simulation of mud-rich subaqueous debris flows on the glacially active margins of the Svalbard–Barents Sea. *Mar. Geol.* 188, 351–364. (doi:10.1016/S0025-3227(02)00310-9)

[59] Elverhøi A, Breien H, De Blasio FV, Harbitz CB, Pagliardi M 2010 Submarine landslides and the importance of the initial sediment composition for run-out length and final deposit. *Ocean Dyn.* 60, 1027–1046. (doi:10.1007/s10236-010-0317-z)

[60] Lastras G, De Blasio FV, Canals M, Elverhøi A 2005 Conceptual and numerical modeling of the BIG’95 debris flow, Western Mediterranean sea. *J. Sedimentary Res.* 75, 784–797. (doi:10.2110/jsr.2005.063)

[61] Elverhøi A, de Blasio FV, Butt FA, Issler D, Harbitz C, Engvik L, Solheim A, Marr J 2002 Submarine mass-wasting on glacially-influenced continental slopes: processes and dynamics. *Geol. Soc. Special Publ.* 203, 73–87. (doi:10.1144/GSL.SP.2002.203.01.05)

[62] Locat J, Lee HJ 2005 Subaqueous debris flows. In *Debris-flow hazards and related phenomena* (eds M Jakob, O Hungr), pp. 203–245. Berlin, Heidelberg, Germany: Springer.

[63] Vanneste M, Harbitz C, De Blasio FV, Glimsdal S, Mienert J, Elverhøi A 2010 *Hinlopen–Yermak landslide, arctic ocean–geomorphology,landslide dynamics, and tsunami simulations.* SEPM Special Publication, no. 95. Tulsa, OK: SEPM.

[64] Iverson RM 1997 The physics of debris flows. *Rev. Geophys.* 35, 245–296. (doi:10.1029/97RG00426)

[65] Elverhøi A, Harbitz C, Dimakis P, Mohrig D, Marr J, Parker G 2000 On the dynamics of subaqueous debris flows. *Oceanography* 13, 109–117. (doi:10.5670/oceanog.2000.20)

[66] De Blasio FV, Breien H, Elverhøi A 2011 Modelling a cohesive-frictional debris flow: an experimental, theoretical, and field-based study. *Earth Surf. Process. Landforms* 36, 753–766. (doi:10.1002/esp.2101)

[67] Loeppky JL, Sacks J, Welch WJ 2009 Choosing the sample size of a computer experiment: a practical guide. *Technometrics* 51, 366–376. (doi:10.1198/TECH.2009.08040)

[68] Tappin DR, Watts P, Grilli ST 2008 The Papua New Guinea tsunami of 17 July 1998: anatomy of a catastrophic event. *Nat. Hazards Earth Syst. Sci.* 8, 243–266. (doi:10.5194/nhess-8-243-2008)

[69] Okal EA, Synolakis CE 2003 A theoretical comparison of tsunamis from dislocations and landslides. *Pure Appl. Geophys.* 160, 2177–2188. (doi:10.1007/s00024-003-2425-x)

[70] Okal EA, Synolakis CE 2004 Source discriminants for near-field tsunamis: near-field tsunamis. *Geophys. J. Int.* 158, 899–912. (doi:10.1111/j.1365-246X.2004.02347.x)

[71] Haugen KB, Løvholt F, Harbitz CB 2005 Fundamental mechanisms for tsunami generation by submarine mass flows in idealised geometries. *Mar. Petroleum Geol.* 22, 209–217. (doi:10.1016/j.marpetgeo.2004.10.016)

[72] Lophaven SN, Nielsen HB, Sondergaard J 2002 DACE- A MATLAB kriging toolbox, version 2.0. IMM-REP, Technical Report.

